# Metallic phase enabling MoS_2_ nanosheets as an efficient sonosensitizer for photothermal-enhanced sonodynamic antibacterial therapy

**DOI:** 10.1186/s12951-022-01344-6

**Published:** 2022-03-15

**Authors:** Huizhi Chen, Xiaojun He, Zhan Zhou, Zhikang Wu, Hai Li, Xinsheng Peng, Yubin Zhou, Chaoliang Tan, Jianliang Shen

**Affiliations:** 1 Guangdong Provincial Key Laboratory of Research and Development of Natural Drugs, and School of Pharmacy, Guangdong Medical University, Dongguan, 523808 China; 2 School of Ophthalmology and Optometry, School of Biomedical Engineering, Wenzhou Medical University, Wenzhou, 325035 Zhejiang China; 3 College of Chemistry and Chemical Engineering, Henan Key Laboratory of Function-Oriented Porous Materials, Luoyang Normal University, Luoyang, 471934 China; 4 Institute of Advanced Materials (IAM) and Key Laboratory of Flexible Electronics (KLoFE), Nanjing Tech University (NanjingTech), 30 South Puzhu Road, Nanjing, 211816 China; 5 Department of Electrical Engineering, City University of Hong Kong, 83 Tat Chee Avenue, Kowloon, Hong Kong China; 6 Shenzhen Research Institute, City University of Hong Kong, Shenzhen, 518057 China; 7 Wenzhou Institute, University of Chinese Academy of Sciences, Wenzhou, 325001 Zhejiang China

**Keywords:** Two-dimensional, Metallic MoS_2_ nanosheets, 1T phase, Sonodynamic property, Antibacterial therapy

## Abstract

Two-dimensional (2D) transition metal dichalcogenide (TMD) nanosheets (e.g., MoS_2_) with metallic phase (1T or 1T´ phase) have been proven to exhibit superior performances in various applications as compared to their semiconducting 2H-phase counterparts. However, it remains unclear how the crystal phase of 2D TMD nanosheets affects their sonodynamic property. In this work, we report the preparation of MoS_2_ nanosheets with different phases (metallic 1T/1T´ or semiconducting 2H) and exploration of its crystal-phase effect on photothermal-enhanced sonodynamic antibacterial therapy. Interestingly, the defective 2D MoS_2_ nanosheets with high-percentage metallic 1T/1T´ phase (denoted as M-MoS_2_) present much higher activity towards the ultrasound-induced generation of reactive oxygen species (ROS) as compared to the semiconducting 2H-phase MoS_2_ nanosheets. More interestingly, owing to its metallic phase-enabled strong absorption in the near-infrared-II (NIR-II) regime, the ultrasound-induced ROS generation performance of the M-MoS_2_ nanosheets can be further enhanced by the photothermal effect under a 1064 nm laser irradiation. Thus, after modifying with polyvinylpyrrolidone, the M-MoS_2_ nanosheets can be used as an efficient sonosensitizer for photothermal-enhanced sonodynamic bacterial elimination under ultrasound treatment combining with NIR-II laser irradiation. This study demonstrates that metallic MoS_2_ nanosheets can be used as a promising sonosensitizer for antibacterial therapy, which might be also promising for cancer therapies.

**Graphical Abstract:**

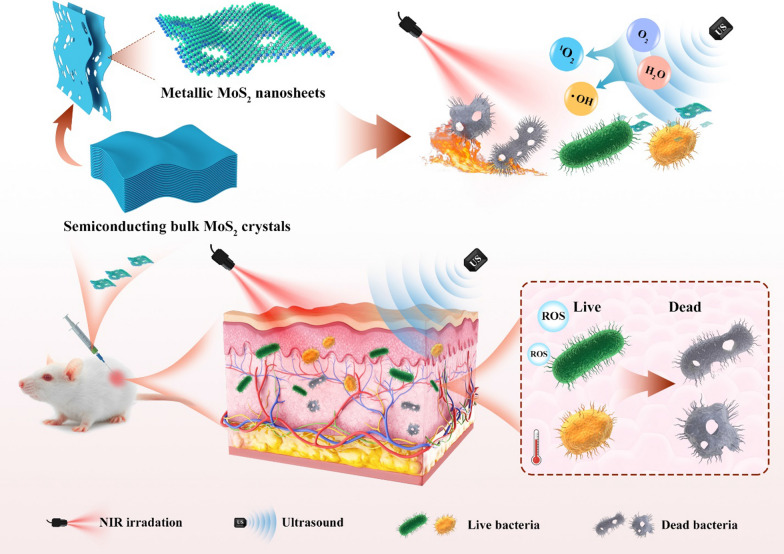

**Supplementary Information:**

The online version contains supplementary material available at 10.1186/s12951-022-01344-6.

## Introduction

The continuously increasing number of multidrug-resistant (MDR) bacteria has been considered as one of the major concerns in global public heath since MDR bacteria significantly limit the therapeutic efficacy of antibiotics and thus yield a high mortality [1–3]. As a consequence, great effort has been devoted to the development of alternative approaches to achieve highly efficient antibacterial performance with negligible resistance concerns [4–11]. Promisingly, sonodynamic therapy has been demonstrated to be an appealing strategy to against MDR bacteria with no resistance concern since a sonosensitizer for sonodynamic therapy can convert the ultrasound (US) energy to generate reactive oxygen species (ROS) [12, 13], which can eliminate various MDR bacteria efficiently [14]. Importantly, the sonodynamic therapy presents excellent tissue-penetrating capability and great biosafety in comparison with other widely explored approaches, such as photothermal therapy and photodynamic therapy, since the US is nonradioactive and has high tissue penetration capability [15]. Developing highly active sonosensitizers is a key step to achieve highly efficient sonodynamic therapy since the sonodynamic therapy is highly dependent on the activity of the sonosensitizer. So far, some inorganic nanomaterials, including TiO_2_ nanoparticles [16], Au@BaTiO_3_ [17], metal ion-doped BiOBr [18, 19], Pt@Pt-T790 [20], and Cu single atoms [21], have developed as sonosensitizers for sonodynamic bacterial elimination. However, the reported sonosensitizers based on inorganic nanomaterials ether exhibited relatively low efficiency or required complicated synthetic procedures. Therefore, developing novel inorganic nanomaterial-based sonosensitizers with superior activity and simple synthetic procedure is still urgent.

Thanks to the rapid development in the field of phase engineering of nanomaterials [22], the crystal phase of two-dimensional (2D) materials, which is determined by their atomic arrangements and/or coordination modes, has been proven to possess a significant impact on the physicochemical properties and application performance in recent years [23–25]. In particular, 2D transition metal dichalcogenides (TMDs) with 2H, 1T, 1T′ and 3R phases, specially MoS_2_ nanosheets, have shown much distinctive properties and performance towards various applications [22, 26]. As a typical example, metallic MoS_2_ nanosheets with 1T, 1T′ or mixed 1T/1T′ phase have shown much enhanced performance in electrocatalysis [27–29], electronic devices [30–32], and energy storage [33–35] in comparison with semiconducting 2H-phase counterparts. Importantly, we have first demonstrated recently that when used as a photothermal nanoagent, the metallic 1T-phase MoS_2_ nanodots present much superior performance in photothermal cancer therapy in the near-infrared (NIR)-II window (1000–1350 nm) in contrast to the 2H-phase counterpart [36]. Our study has proven the great potential of metallic TMDs in biomedical applications. However, how the crystal phase of TMDs affects their sonodynamic properties and antibacterial performance still remains unclear. Moreover, no report has been found on the utilization of 2D TMDs as sonosensitizers for sonodynamic-related biomedical applications.

In this contribution, we prepare 2D MoS_2_ nanosheets with different phases (metallic 1T/1T´ and semiconducting 2H) and explore their crystal phase-dependent performance as sonosensitizers for photothermal-enhanced sonodynamic antibacterial therapy. It was found that metallic 1T/1T´-phase could endow the defective 2D metallic MoS_2_ (denoted as M-MoS_2_) with much enhanced activity when used as a sonosensitizer for the US-induced generation of ROS as compared to the semiconducting 2H-phase MoS_2_ nanosheets (denoted as S-MoS_2_). Importantly, the excellent photothermal effect of the M-MoS_2_ nanosheets enabled by its metallic phases (1T/1T´ phase) could further enhance its US-induced ROS generation capability though a NIR-II laser (1064 nm) irradiation. Thus, after surface modification with polyvinylpyrrolidone (PVP), the M-MoS_2_ nanosheets can be used as an efficient sonosensitizer for bacterial elimination.

## Material and methods

### Chemicals

Semiconducting 2H-phase MoS_2_ bulk crystals and *n*-butyllithium (2.0 M in cyclohexane) were purchased from Sigma-Aldrich (USA). Hexane (AR) was purchased from Tianjin Damao Chemical Reagent Co., Ltd. (China). 1,3-Diphenylisobenzofuran (DPBF, 98%), Rhodamine B (RB, 98%) and o-phenylenediamine (98%) were obtained from Energy Chemical Co., Ltd. (China). No further purification was conducted on all the chemicals. All the water used in our experiments was purified by a Milli-Q System (Millipore).

### Preparation of M-MoS_2_ nanosheets

The M-MoS_2_ nanosheets were prepared from the semiconducting 2H-phase MoS_2_ bulk crystals by the previously reported chemical Li-intercalation method with slight modifications [37]. The MoS_2_ bulk crystals (100 mg) were dispersed in 10 mL of *n*-butyllithium solution (2 M in cyclohexane) for 4 days in a glove box to obtain Li-intercalated material. The Li-intercalated material was then washed three times with hexane after removing the upper *n*-butyllithium solution carefully. Then, the Li-intercalated material was dispersed in 100 mL water and sonicated for 30 min under the ice bath environment to obtain a uniform dispersion. After removing the large-size nanosheets by centrifugation (3000 r.p.m for 10 min), the suspension was centrifuged at 8000 r.p.m for another 10 min and then washed with DI water to collect the M-MoS_2_ nanosheets.

### Preparation of S-MoS_2_ nanosheets

The S-MoS_2_ nanosheets were prepared by the phase transformation of the M-MoS_2_ nanosheets via the hydrothermal method according to the previous report [38]. Briefly, the aqueous suspension of M-MoS_2_ nanosheets (0.2 mg mL^−1^) was added into a glass vial sealed with a latex plug, and then the high purity nitrogen gas was pumped into the solution to remove oxygen for 8 h. After transferring the gas vial into a nitrogen glove box, the latex plug was opened to further deoxygenate the solution. The above solution was transferred into a hydrothermal reaction vessel and heated to 210 °C. After heating for 2 h, the reactor was cooled to room temperature naturally. The suspension of S-MoS_2_ nanosheets was obtained after washing with DI water via centrifugation.

### Characterization

A transmission electron microscope (JEOL JEM-2100F) was used to record TEM images. A transmission electron microscope (JEOL ARM200F) with double hexapole Cs correctors (Heidelberg, Germany) was used to take atomic-resolution STEM images. Powder XRD patterns were measured on a Bruker D8 diffractometer (German), in which a Cu Kα (λ = 1.54178 Å) is used as the X-ray source. A Dimension ICON with Nanoscope V controller (Bruker) was used to perform tapping mode AFM measurements under ambient conditions. XPS spectra were recorded on a Thermo Scientific K-Alpha + instrument and calibrated by using the C1s peak as the reference. A HITACHI UH5300 spectrometer was used to measure the UV–Vis-NIR absorption spectra. The concentration of metal elements was analysed by an inductively coupled plasma-optical emission spectrometry (Agilent 5110).

### Photothermal effects of M-MoS_2_ and S-MoS_2_ nanosheets

To investigate the photothermal effects of M-MoS_2_ and S-MoS_2_ nanosheets under a 1064 nm continuous laser irradiation, samples were placed in tubes with 100 μL of DI water and were treated under a 1064 nm laser irradiation (1 W cm^−2^). The surface temperature changes were monitored by a thermal imager (E4, FLIR, USA).

### ROS generation by US activation

The 1,3-diphenylisobenzofuran (DPBF) has been widely used as a typical molecular probe for the detection of singlet oxygen (^1^O_2_) generation. In a typical process, 20 μg mL^−1^ of DPBF and 50 μg mL^−1^ of M-MoS_2_ and S-MoS_2_ nanosheets were dispersed in 3.0 mL phosphate buffer saline (PBS, 0.1 M, pH 7.4). After different US irradiation (1.0 MHz, 1.5 W cm^−2^, 50% duty cycle) durations, the absorbance changes of DPBF at 416 nm were recorded using UV–Vis-NIR spectroscopy (CARY 5000, Agilent Technologies, USA) to quantify the generation rate of ROS by M-MoS_2_ and S-MoS_2_ nanosheets.

### Antibacterial in vitro

To investigate the antibacterial properties of samples in vitro, both *Staphylococcus aureus* (*S. aureus* strain, ATCC 29,213) and *Pseudomonas aeruginosa* (*P. aeruginosa*, PAO1) were selected. In a typical process, the bacterial (*S. aureus* / *P. aeruginosa*) with a final concentration of 10^7^ CFU mL^−1^ was added into the PBS, PVP-modified M-MoS_2_ and S-MoS_2_ nanosheets solution (50 ppm), respectively. Then, the above solution was placed in the dark, or irradiated by a 1064 nm NIR laser, a 1.0 MHz of US (1.5 W cm^−2^, 50% duty cycle), or NIR Laser + US for three minutes, respectively. The bacteria with different treatments were diluted to 1000X by PBS, followed by transfer of 20 μL into the Tryptone Soy Broth (TSB) plate and incubated at 37 °C for 12 h. Finally, the colonies of bacteria in the plates were counted in triplicate for all experimental groups.

The morphologies of *S. aureus* strain treated under different conditions were explored by a SEM (SU8010, Hitachi, Japan). After treating with the different crystal phases of MoS_2_ nanosheets, the *S. aureus* strain was fixed by the 2.5% of glutamate, followed by washing with PBS for twice. Furthermore, the bacteria were dehydrated successively by ethanol solutions in gradient concentrations (70, 50, 30, 10, and 0% v/v) for 15 min, and then dropped onto the silicon wafers and coated on platinum for imaging by SEM.

### Antibacterial and wound healing in vivo

All animal experiments were monitored and approved by the Committee of Wenzhou Medical University. The 6–8 weeks old female mice (BALB/c) were randomly divided into five groups, which were as follows: (1) PBS, (2) M-MoS_2_, (3) M-MoS_2_ + US, (4) M-MoS_2_ + Laser, (5) M-MoS_2_ + US + Laser (n = 5 per group). The concentration of M-MoS_2_ nanosheets was 50 μg mL^−1^. To construct the mice infection model, 50 µL of 10^9^ CFU mL^−1^ *S. aureus* bacteria solution was injected subcutaneously into the both sides of the spine. One day later, abscesses were formed and irradiated by NIR laser (1064 nm) and US for 3 min after anesthesia by chloral hydrate (10 mg/g, 4% w/w). After 10 days of different treatments, the infectious tissues in all groups were collected and cultured into the TSB plate for colony counting. Furthermore, the mice were euthanized by chloral hydrate, and their main organs (heart, liver, spleen, lung, and kidney) and skin tissues were treated by paraformaldehyde solution (4%) for H&E, Gram and Masson trichrome staining.

## Results and discussion

**Fig. 1:**
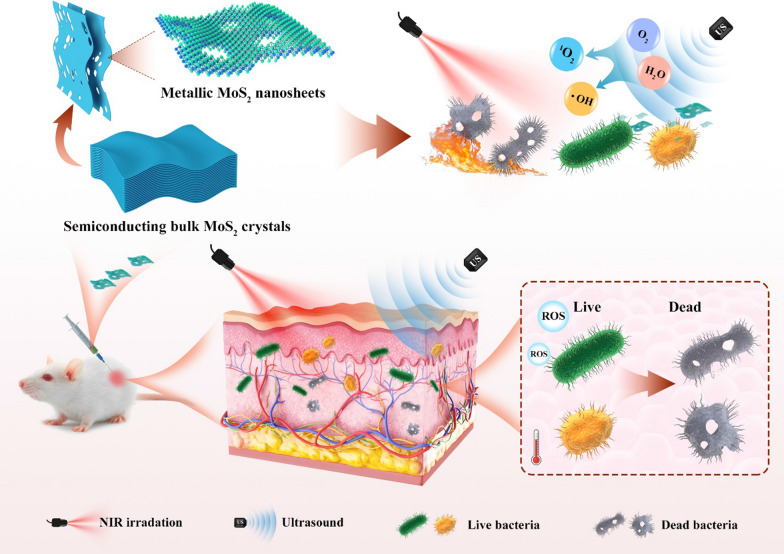
Schematic demonstration of the liquid exfoliation of semiconducting bulk MoS_2_ crystals to obtain defective metallic MoS_2_ nanosheets and its usage as an efficient agent for photothermal-enhanced sonodynamic antibacterial application

Ultrathin metallic 2D MoS_2_ nanosheets, i.e., M-MoS_2_, were first prepared by exfoliating 2H-phase MoS_2_ bulk crystals via the previously reported chemical Li-intercalation method with slight modifications [37]. As revealed by the scanning electron microscopy (SEM) images (Additional file 1: Fig. S1a), the commercial 2H-phase MoS_2_ bulk crystals have a plate-like morphology with a lateral size of 10–50 μm and thickness of 1–2 μm. All the peaks from the X-ray diffraction (XRD) pattern of 2H-phase MoS_2_ bulk crystals match well with the simulated 2H-phase reference, confirming its 2H phase crystal structure. As for the exfoliation, 2H-phase MoS_2_ bulk crystals were first immersed in the *n*-butyllithium solution for 4 days to form Li-intercalated compounds. Here the immersion time in the *n*-butyllithium solution was prolonged from 2 to 4 days to generate more defects on the basal plane of the obtained M-MoS_2_ nanosheets. Note that the intercalation of lithium ions into MoS_2_ bulk crystals can induce the phase transformation from 2H phase to metallic 1T/1T´ phase. As a consequence, after taken out and then sonication in water, water-dispersed defective M-MoS_2_ nanosheets can be obtained (Fig. 1). Interestingly, through a simple hydrothermal treatment under nitrogen atmosphere, the M-MoS_2_ nanosheets can be transformed back into semiconducting 2H-phase MoS_2_ nanosheets, i.e., S-MoS_2_, without obvious structure changes [38].

**Fig. 2:**
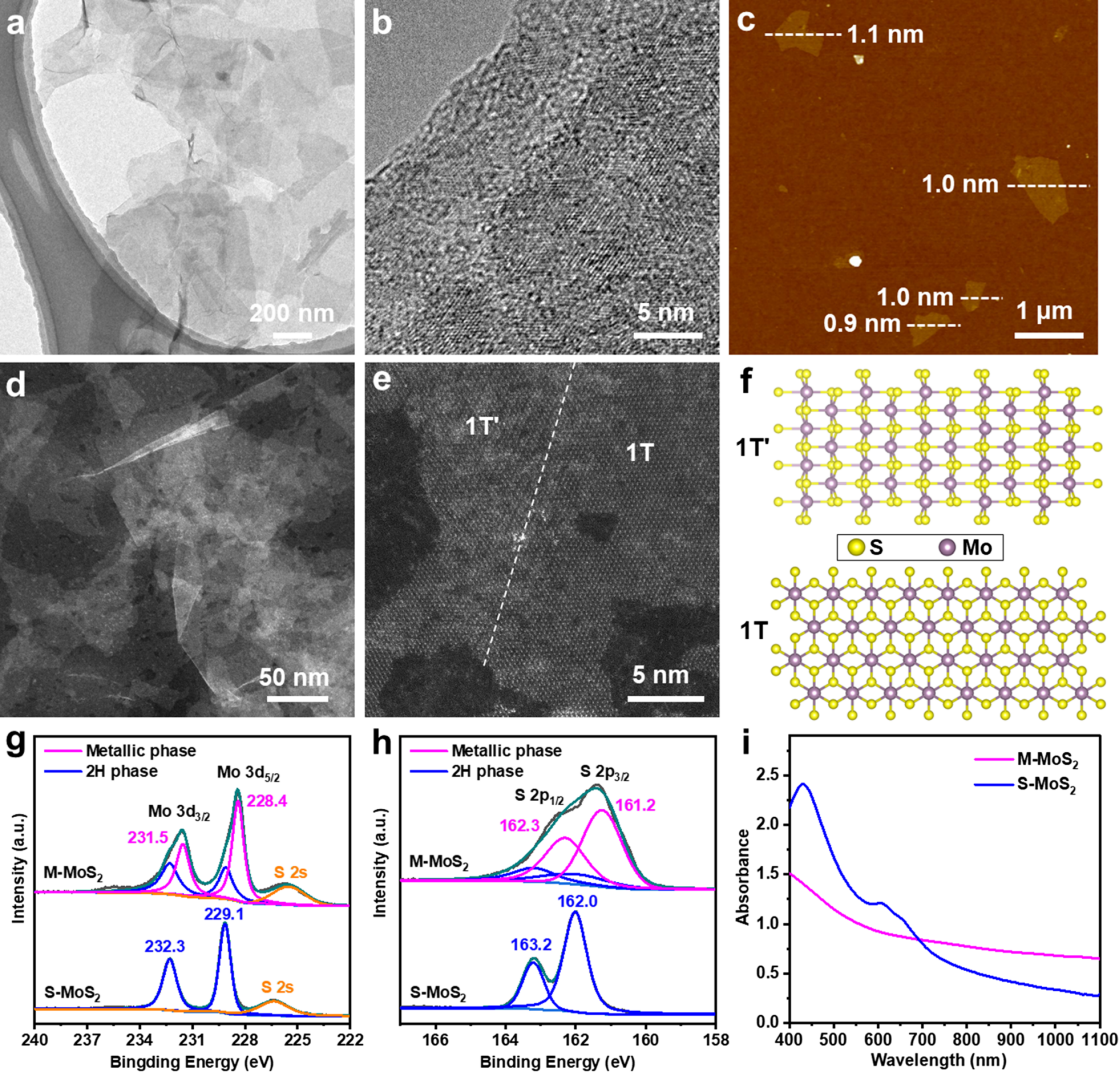
**a** TEM and **b** HRTEM images of M-MoS_2_ nanosheets. **c** AFM height image of M-MoS_2_ nanosheets. **d** HAADF-STEM of M-MoS_2_ nanosheets and **e** the atomic-resolution HAADF-STEM of a M-MoS_2_ nanosheet. **f** The crystal structures of 1T-phase and 1T´-phase MoS_2_. High-resolution XPS **g** Mo 3d and **h** S 2p spectra of M-MoS_2_ and S-MoS_2_ nanosheets. **i** UV–Vis-NIR spectra of 35 ppm solution of M-MoS_2_ and S-MoS_2_ nanosheets

The characterization of defective 2D M-MoS_2_ nanosheets is shown in Fig. 2. The transmission electron microscopy (TEM) image shows that the M-MoS_2_ nanosheets have a size ranging from hundreds of nanometres to around one micrometre (Fig. 2a). The ultra-low contrast of the M-MoS_2_ nanosheets proves its ultrathin thickness. The high-resolution TEM (HRTEM) image of a typical M-MoS_2_ nanosheet shows a continuous lattice fringe (Fig. 2b), suggesting its crystalline structure. The thickness of M-MoS_2_ nanosheets was characterized by atomic force microscope (AFM). As evidenced by its AFM height image (Fig. 2c), the measured thickness of the M-MoS_2_ nanosheets is ~ 0.9–1.1 nm, proving that the M-MoS_2_ nanosheets are single-layer. The M-MoS_2_ nanosheets were further characterized by scanning transmission electron microscopy (STEM). As shown in Fig. 2d, the low-magnification STEM image shows the ultrathin sheet morphology of M-MoS_2_ nanosheets with a lateral size of hundreds of nanometres. Of note that benefiting from the excellent contrast, rich hole defects can be clearly observed on the basal plane of M-MoS_2_ nanosheets from the STEM image (Fig. 2d). The atomic resolution STEM image of a typical M-MoS_2_ nanosheet is shown in Fig. 2e. Both the 1T (right side) and 1T´ (left side) phases can be clearly identified from the atomic resolution STEM image (Fig. 2e). Because of the dislocation of the two S atoms between two nearest Mo atoms and the small Z number of S atoms, the S atoms show negligible contrast in atomic resolution STEM image. The STEM results are consistent with the corresponding crystal structures of 1T- and 1T´-phase (Fig. 2f). Importantly, small hole-like defect sites can be also observed on the M-MoS_2_ nanosheet. The aforementioned structural analysis strongly supports that the as-prepared M-MoS_2_ nanosheets contain mixed metallic 1T/1T´ phase and rich defects. Owning to their metastable nature, metallic 1T/1T´ phases could be changed back to semiconducting 2H phase by proper treatments, such as thermal annealing. Therefore, semiconducting 2H-phase MoS_2_ nanosheets, i.e., S-MoS_2_, were prepared by the hydrothermal treatment of M-MoS_2_ nanosheets under nitrogen atmosphere. The characterization of S-MoS_2_ nanosheets is shown in Additional file 1: Fig. S2. The TEM image shows that the S-MoS_2_ nanosheets well maintain the sheet-morphology with a lateral size of few hundreds of nanometres (Additional file 1: Fig. S2a). As shown in Additional file 1: Fig. S2b, a continuous lattice fringe can be observed from the HRTEM image of a typical S-MoS_2_ nanosheet, suggesting its crystalline structure. The AFM height image shows that the thickness of the S-MoS_2_ nanosheets is ranging from 1.5 to 3.9 nm (Additional file 1: Fig. S2c), revealing that they are few-layer thick. Such result suggests that stacking of monolayers also happened during the hydrothermal treatment process to form few-layer S-MoS_2_ nanosheets. The atomic resolution STEM image and its corresponding filtered image of S-MoS_2_ nanosheets show a hexagonal lattice arrangement (Additional file 1: Fig. S2d,e), which is consistent with the crystal structure of 2H-phase MoS_2_ (Additional file 1: Fig. S2f), confirming the phase transformation from metallic 1T/1T´ to 2H phase of MoS_2_ nanosheets.

Both the M-MoS_2_ and S-MoS_2_ nanosheets were further characterized by X-ray photoelectron spectroscopy (XPS) and UV–vis-NIR absorption spectroscopy. As shown in the high-resolution XPS Mo 3d spectrum (Fig. 2g), the M-MoS_2_ nanosheets give two main peaks at 231.5 eV and 228.4 eV, which are assignable to metallic 1T/1T´ phase [39]. It is worth pointing out that 1T and 1T´ phase cannot be distinguished from each other from XPS spectra. The M-MoS_2_ nanosheets also show two shoulder peaks at 232.3 eV and 229.1 eV (Fig. 2g), which are assignable to the 2H phase [39]. In contrast, the S-MoS_2_ nanosheets only display two 2H-phase peaks at 232.3 eV and 229.1 eV (Fig. 2g) [39]. Similarly, the high resolution XPS S 2p spectrum of the M-MoS_2_ nanosheets also shows two major peaks at 162.3 and 161.2 eV corresponding to metallic 1T/1T´ phase along with two shoulder 2H-phase peaks at 163.2 and 162.0 eV [39]. In contrast, the high resolution XPS S 2p spectrum of the S-MoS_2_ nanosheets only present two peaks at 163.2 and 162.0 eV, assignable to the 2H phase [39]. As evidenced by the XPS characterization, metallic 1T/1T´ phases are the dominant phase in the M-MoS_2_ nanosheets and the calculated percentage based on the Mo 3d spectrum is ~ 70%. Such result is quite consistent with previously reported metallic MoS_2_ nanosheets prepared by a similar method [27, 39]. The metallic or semiconducting nature of the prepared MoS_2_ nanosheets is further verified by the absorption spectra. As shown in Fig. 2i, the M-MoS_2_ nanosheets shows a continuously and slowly decreased absorption spectrum without any characteristic absorption peaks from 400 to 1100 nm, suggesting its metallic feature. In contrast, the S-MoS_2_ nanosheets display three characteristic peaks at 427, 606 and 653 nm (Fig. 2i) [39]. It is worth mentioning that the M-MoS_2_ nanosheets show decent absorption intensity in comparison with the negligible absorption of the S-MoS_2_ nanosheets in the NIR regime, which is similar to our previously reported MoS_2_ nanodots with 1T or 2H phase [36].

Owing to its strong NIR absorption, the M-MoS_2_ nanosheets could be used as a photothermal agent. To this end, we first measured the concentration-dependent UV–vis-NIR absorption spectra of M-MoS_2_ nanosheets (Additional file 1: Fig. S3a) and the calculated its mass extinction coefficient at 1064 nm (Additional file 1: Fig. S3b), which is ~ 26.4 L g^−1^ cm^−1^. Such value is comparable with the mass extinction coefficient (25.6 L g^−1^ cm^−1^) of our previously reported 1T-phase MoS_2_ nanodots [36]. Similarly, we modified the M-MoS_2_ and S-MoS_2_ nanosheets with PVP to enhance their physiological stability and biocompatibility. Thereafter, the photothermal properties of PVP-modified M-MoS_2_ and PVP-modified S-MoS_2_ nanosheets were studied using a NIR-II laser at 1064 nm. As shown in Additional file 1: Fig. S4a, the NIR thermal photos of PVP-modified M-MoS_2_ solution display that temperature increased and significantly improved with the increasing concentration (from 0 to 50 ppm) under the irradiation by a 1064 nm laser at 1.0 W cm^−2^ for 6 min, while the PVP-modified S-MoS_2_ solution show negligible temperature change at the same condition. The PVP-modified M-MoS_2_ solution could quickly heat up under the irradiation by a 1064 nm laser (1.0 W cm^−2^), and its temperature increased to 58.9 ℃ at a low concentration (50 ppm) in 3 min then gradually reached a stable condition, while the temperature of pure water did not change significantly (Additional file 1: Fig. S4b). As for the thermal curves of PVP-modified S-MoS_2_ solution, its temperature only increased to 35.8 ℃ after treating at the same conditions for 3 min (Additional file 1: Fig. S4c). Such results indicate the excellent photothermal property of the M-MoS_2_ nanosheets, superior to that of the S-MoS_2_ nanosheets. As shown in photothermal heating curves of PVP-modified M-MoS_2_ (50 ppm) irradiated with a laser at different power densities (Additional file 1: Fig. S4d), its temperature was raised to 48 ℃ even under low-power irradiation at 0.5 W cm^−1^ for 3 min. Promisingly, both PVP-modified M-MoS_2_ and S-MoS_2_ nanosheets displayed excellent photothermal stability after five On/Off cycles by a 1064 nm laser irradiation (Additional file 1: Fig. S4e).

**Fig. 3:**
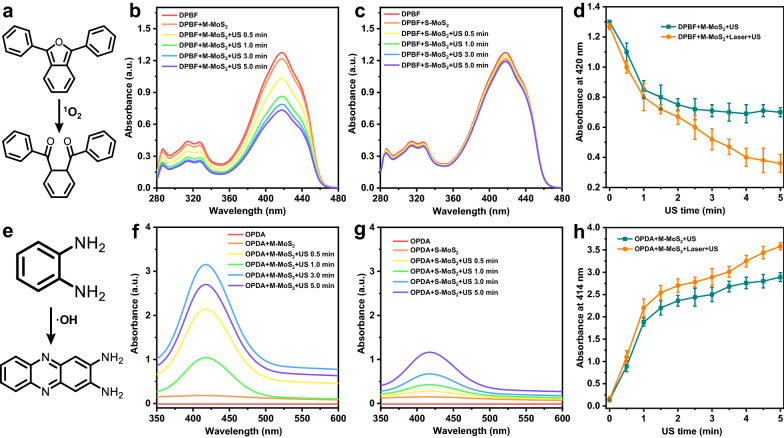
**a** The molecular structure of the reaction between DPBF and active oxygen species. **b**, **c** UV–vis spectra of DPBF in absence of and in presence of 50 ppm **b** M-MoS_2_ and **c** S-MoS_2_ nanosheets after US treatments with different times. **d** The comparison of the SD performance of M-MoS_2_ nanosheets with and without 1064 nm laser irradiation measured by DPBF. **e** The molecular structure of the reaction between OPDA and active oxygen species. **f**, **g** UV–vis spectra of OPDA in absence of and in presence of 50 ppm **f** M-MoS_2_ and **g** S-MoS_2_ nanosheets after US treatments with different times. **h** The comparison of the SD performance of M-MoS_2_ nanosheets with and without 1064 nm laser irradiation measured by OPDA

In addition, we also explored the crystal phase-dependent sonodynamic performance of MoS_2_ nanosheets used as sonosensitizers for sonodynamic therapy. The PVP-modified M-MoS_2_ and S-MoS_2_ nanosheets were used as sonosensitizers to degrade the classic organic dye (Rhodamine B: RB) under the US treatment. As shown in Fig.S5a, RB has greater rigidity and conjugate structure, making it have strong absorption in the visible light region. Upon exposing to PVP-modified M-MoS_2_ for 3 min under ultrasonic treatment (1.0 MHz, 1.5 W cm^−2^, 50% duty cycle), the relative intensity of the UV absorption at 564 nm of RB was dramatically reduced by 60.6% (Additional file 1: Fig. S5b), while the intensity only decreased by 14.3% for the PVP-modified S-MoS_2_ (Additional file 1: Fig. S5c), demonstrating that the 2D M-MoS_2_ nanosheets can generate more ROS than that of the S-MoS_2_ nanosheets under the ultrasonic treatment. To further understand the specific types of ROS, 1,3-diphenylisobenzofuran (DPBF) and *O*-phenylenediamine (OPDA) were used as the probe to monitor the generation of singlet oxygen (^1^O_2_) and hydroxyl radicals (·OH) from the MoS_2_ nanosheets by the US treatment, respectively (Fig. 3). The UV absorption band (from 280 to 480 nm) of DPBF with the PVP-modified M-MoS_2_ decreased rapidly with the prolongation of US irradiation durations (Fig. 3b), while continuous US treatment on the DPBF solution with the PVP-modified S-MoS_2_ only induced slight changes on the DPBF absorption (Fig. 3c). Such result suggests that the PVP-modified M-MoS_2_ nanosheets could continuously generate ^1^O_2_ from water by trigging with US. Furthermore, OPDA could be oxidized by ·OH to produce the yellow product that display the characteristic peak at 414 nm. As shown in Fig. 2f,g, the characteristic peak of OPDA appeared and was significantly enhanced after exposing to the PVP-modified M-MoS_2_ nanosheets under US irradiation in contrast to the slightly enhancement after exposing to the PVP-modified S-MoS_2_ nanosheets, suggesting that more ·OH will be generated by the M-MoS_2_ nanosheets than that of the S-MoS_2_ nanosheets. All the aforementioned results proved that the defective M-MoS_2_ nanosheets can be a highly efficient sonosensitizer for the US-induced generation of ROS, which is much superior than that of the S-MoS_2_ nanosheets. Importantly, the activity toward the US-induced generation of ROS of the M-MoS_2_ nanosheets can be further enhanced by its excellent photothermal effect. The PVP-modified M-MoS_2_ as a sonosensitizer could produce more ROS (^1^O_2_ and ·OH) under the combination of US and NIR laser irradiation (Fig. 3d and 3h).

We believed that both the excellent photothermal and photodynamic properties of the M-MoS_2_ nanosheets are attributed to the metallic nature of metallic 1T/1T´ phases. First, the metallic nature of metallic 1T/1T´ phases endows the M-MoS_2_ nanosheets with strong absorption in the NIR-II regime because of its zero-bandgap structure [40], thus enabling its excellent photothermal performance under NIR-II laser irradiation. Second, the metallic nature of metallic 1T/1T´ phases endows the M-MoS_2_ nanosheets with excellent activity towards the ROS generation induced by US treatment because of the zero-bandgap structure. Previous studies have proven that inorganic nanomaterials with smaller bandgaps are easier to be activated to generate ROS under external stimuli since they need less energy to achieve electron excitation [41, 42]. In contrast, the S-MoS_2_ nanosheets with 2H phase have a large bandgap around 1.8 eV [43]. Frist, the large bandgap of 2H phase makes the S-MoS_2_ nanosheets unable to absorb the light in the NIR regime, thus giving rise to poor photothermal performance under NIR laser irradiation. Second, the large bandgap of 2H phase makes the S-MoS_2_ nanosheets also makes it need much more energy to activate the ROS generation, thus yielding poor activity towards the ROS generation under US treatment. That is why the M-MoS_2_ nanosheets showed superior performance both in photothermal and photodynamic therapies.

**Fig. 4:**
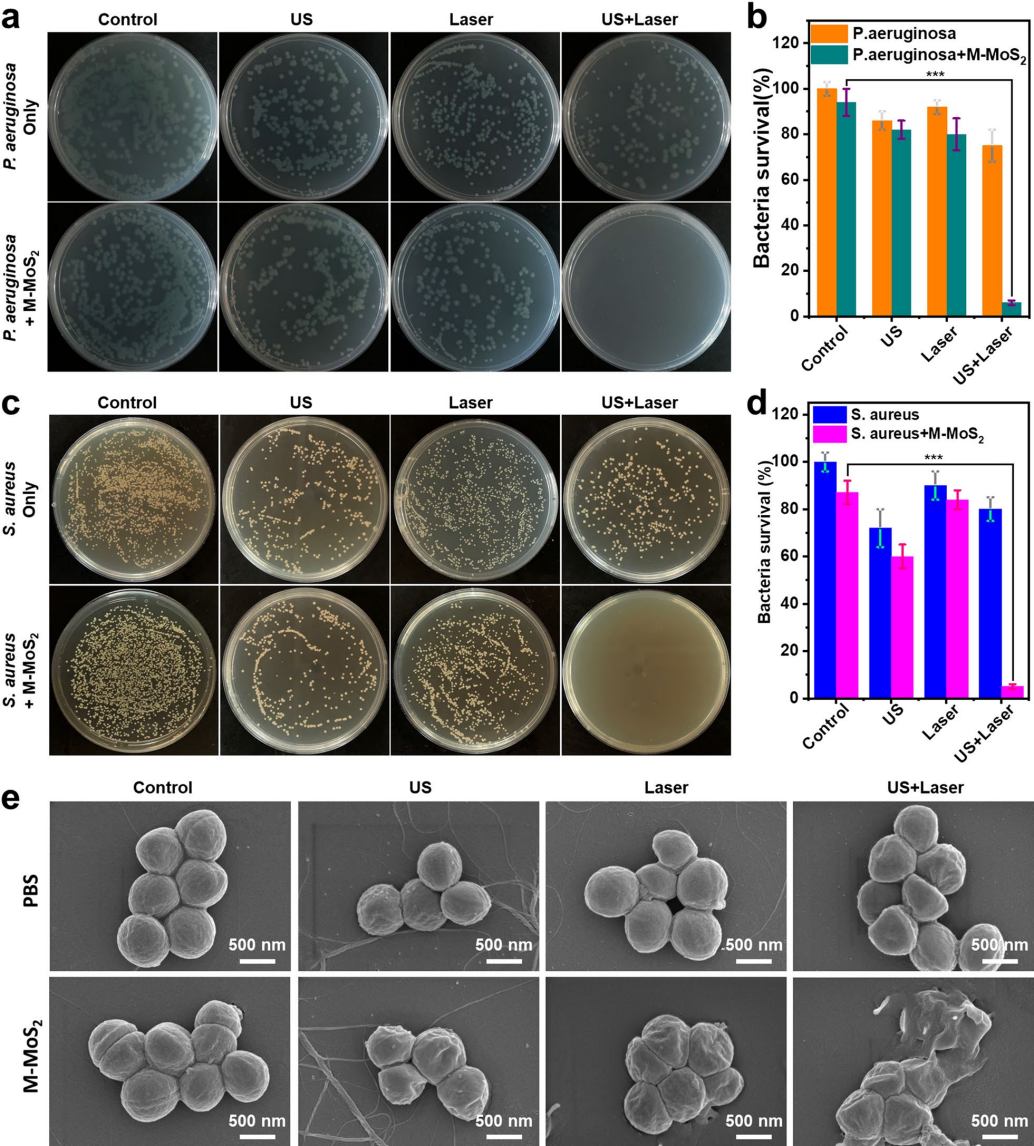
Photographs of bacterial colonies formed by *P. aureus* (**a**) and *S. aureus* (**c**) after treating with PBS (control), PBS + US, PBS + Laser, PBS + US + Laser, M-MoS_2_, M-MoS_2_ + US, M-MoS_2_ + Laser, M-MoS_2_ + US + Laser. **b**, **d** The bacterial survival from the bacterial colonies in **a**, **c** (Student’s two-tailed t-test, ***p < 0.001). **e** The SEM images of *S. aureus* bacterial treated with different treatments

The biocompatibility of a material is very important for its subsequent biological applications. L929 cell line was used to evaluate the cytotoxicity of the PVP-modified M-MoS_2_ and S-MoS_2_ nanosheets by MTT assay. As shown in Additional file 1: Fig. S6, both the PVP-modified M-MoS_2_ and S-MoS_2_ nanosheets displayed low cytotoxicity. Even at high concentration (150 ppm) for 24 h culturing with cells, the cell survival rate remained above 80%. Considering its excellent photothermal effect and sonodynamic performance, the PVP-modified M-MoS_2_ nanosheets can be used as an efficient nanoagent for photothermal-enhanced sonodynamic antibacterial therapy. The antibacterial activity of the PVP-modified M-MoS_2_ nanosheets was explored using *P. aeruginosa* and *S. aureus* by the plate count method. It was obviously decreased the number of colonies for both of M-MoS_2_ + US and M-MoS_2_ + Laser groups in contrast to the control and M-MoS_2_ groups (Fig. 4a and c). After US (1.0 MHz, 1.5 W cm^−2^, 50% duty cycle) and laser (1064 nm, 1 W cm^−2^) irradiation for 3 min, the bacteriostatic rate of PVP-modified M-MoS_2_ (50 ppm) reached 18% and 20% for *P. aeruginosa* (40% and 16% for *S. aureus*), respectively (Fig. 4b and d). More interestingly, group M-MoS_2_ + US + Laser displayed exceedingly effective sterilization performance (almost 100%) both on *P. aeruginosa* and *S. aureus* with the combined treatment of US and light irradiation for 3 min, indicating that the photothermal effect of the M-MoS_2_ nanosheets promoted US-induced ROS to enhance sterilization efficiency. Furthermore, the morphological changes of *S. aureus* were explored using SEM images (Fig. 4e). Cell membranes presented smooth and intact in the PBS group, and no obvious damage could be observed even with the treatment of US or laser irradiation. However, different degrees of wrinkles and destruction were observed on the surface of *S. aureus* in the M-MoS_2_ group after irradiating by US or NIR laser, especially the combined treatment of US and laser. Such results further demonstrate that the M-MoS_2_ nanosheets have excellent photothermal-enhanced sonodynamic antibacterial performance.

**Fig. 5:**
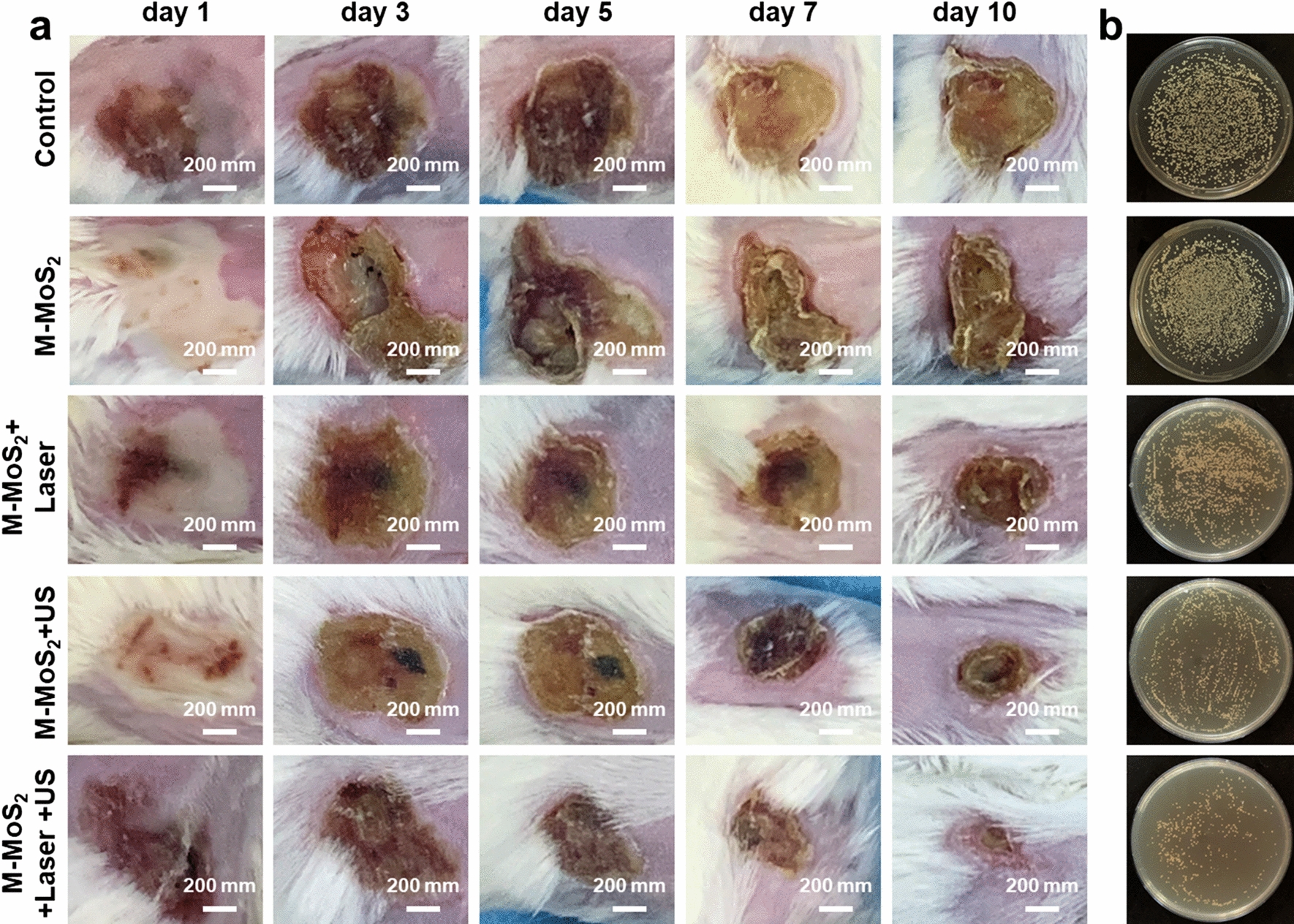
**a** Photographs of the infected wound treated with different treatments (PBS, PBS + US, PBS + Laser, PBS + US + Laser, M-MoS_2_, M-MoS_2_ + US, M-MoS_2_ + Laser, and M-MoS_2_ + US + Laser). **b** The infectious tissues were collected and homogenized in TSB for colony counting after treating with different treatments for 10 days

Encouraged by the above results on the antibacterial assay in vitro, the PVP-modified M-MoS_2_ nanosheets were evaluated as a sonosensitizer in photothermal-enhanced sonodynamic therapy for wound healing in vivo. Firstly, *S. aureus* bacteria solution was injected subcutaneously into the mice to construct the infection model. Mice with infectious wounds were then divided into five groups: control, PVP-modified M-MoS_2_, PVP-modified M-MoS_2_ + Laser, PVP-modified M-MoS_2_ + US, and PVP-modified M-MoS_2_ + Laser + US. Both in the control and PVP-modified M-MoS_2_ groups, large area secretion and the purulent water leakage was observed even after 10 days of healing (Fig. 5a), suggesting serious infection of the wounds. In contrast, the wounds in PVP-modified M-MoS_2_ + Laser and PVP-modified M-MoS_2_ + US groups became smaller and began to scramble at day 10. Interestingly, the mice in the PVP-modified M-MoS_2_ + Laser + US group exhibited the smallest wound area and the wound almost completely disappeared after 10 days of healing. After treating with different treatment for 10 days, the infectious tissues were collected and homogenized in TSB for colony counting. The bacterial survival rate of the PVP-modified M-MoS_2_ group was almost the same as that of the control group, while the bacteria in PVP-modified M-MoS_2_ + Laser + US group had the lowest survival rate (Fig. 5b and Additional file 1: Fig. S7). The bacterial viability was followed by PVP-modified M-MoS_2_ + Laser group and PVP-modified M-MoS_2_ + US group (Fig. 5b and Additional file 1: Fig. S7). These results further indicate that ROS generated by photothermal enhanced US had significant antibacterial application in wound healing.

To evaluate the repair of wounds, the histological sections of the infected tissues from different groups were examined by using Gram, Masson trichrome, and Hematoxylin–Eosin (H&E) staining after treating for 10 days. Gram staining is a technique that can identify the negative and positive bacteria, and the positive *S. aureus* can be stained by blue colour. The blue colour decreased from left to right (Additional file 1: Fig. S8, photos in the first line), suggesting the decrease in the number of gram-positive bacteria, which was consistent with the results of colonies (Fig. 5b). Masson trichrome and H&E staining results indicated that the collagen deposition and the surface flatness were much better in PVP-modified M-MoS_2_ + Laser + US group than other groups (Additional file 1: Fig. S8). Therefore, the prepared PVP-modified M-MoS_2_ nanosheets can be used as a promising sonosensitizer for effective antibacterial and promote wound healing. Interestingly, no obvious pathological abnormalities were observed in the H&E staining of main organs (heart, live, spleen, lung, and kidney) after treating with different ways, demonstrating that PVP-modified M-MoS_2_ with negligible biotoxicity to mice at the dose used (Additional file 1: Fig. S9).

## Conclusions

In summary, we have achieved the preparation of 2D MoS_2_ nanosheets with different phases (1T/1T´ or 2H phase) and explored the impact of the crystal phase on their sonodynamic activity. It was found that owing to its metallic and defect-rich nature, the M-MoS_2_ nanosheets exhibited superior activity towards the US-induced ROS generation than that of the S-MoS_2_ nanosheets. More importantly, the metallic phases can endow the M-MoS_2_ nanosheets with strong absorption in the NIR-II regime and thus the photothermal effect irradiated by a 1064 nm laser can be used to further enhance the US-induced ROS generation performance. After PVP modification, the M-MoS_2_ nanosheets can be used as an efficient sonosensitizer for photothermal-enhanced sonodynamic bacterial elimination under US treatment combining with NIR-II laser irradiation. We believe that further increasing the percentage of metallic phase in 2D MoS_2_ nanosheets is expected to further enhance its sonodynamic performance since ~ 30% of the M-MoS_2_ nanosheets is still 2H phase. This study has first demonstrated that the crystal phase of nanomaterials also has a significant impact on their sonodynamic performance, making metallic MoS_2_ a promising sonosensitizer for antibacterial application.

## Supplementary Information

**Additional file 1**: **Fig. S1** (a) SEM images of 2H-phase MoS_2_ bulk crystals and (b) its XRD pattern with simulated reference. **Fig. S2** (a) TEM image of S-MoS_2_ nanosheets and (b) the HRTEM image of a typical S-MoS_2_ nanosheet. (c) AFM height image of S-MoS_2_ nanosheets. (d) Atomic-resolution HAADF-STEM image of a typical S-MoS_2_ nanosheet. (e) The filtered of the marked squire regime in (d) and (f) the corresponding crystal structure of semiconducting 2H-phase MoS_2_. **Fig. S3** (a) UV–vis-NIR absorption spectra of M-MoS_2_ nanosheets dispersed in water at different concentrations and (b) its corresponding normalized absorbance intensity divided by the characteristic length of the cell (A/L) at different concentrations for λ = 1064 nm. **Fig. S4** (a) Thermal images of PVP-modified M-MoS_2_ and PVP-modified S-MoS_2_ nanosheets with different concentrations (0, 10, 20, and 50 ppm) under the irradiation by a laser at 1.0 W cm^−2^ for 6 min. Photothermal heating curves water solutions containing (b) PVP-modified M-MoS_2_ and (c) PVP-modified S-MoS_2_ nanosheets at different concentrations (0, 5, 10, 20 and 50 ppm). (d) Photothermal heating curves water solutions containing 50 ppm PVP-modified M-MoS_2_ irradiated with a laser at different power density. (e) Heating of solution of 50 ppm PVP-modified M-MoS_2_ and S-MoS_2_ nanosheets for five On/Off cycles. **Fig. S5** (a) The molecular structure of the reaction between RB and active oxygen species. (b,c) UV–vis spectra of RB in absence of and in presence of 50 ppm (b) M-MoS_2_ and (c) S-MoS_2_ nanosheets after US treatments with different times. **Fig. S6** Relative viabilities of cells (L929) after incubation in PVP-modified M-MoS_2_ and S-MoS_2_ nanosheets with different concentrations (0, 5, 10, 20, 40, 80, 100, 120, 150 and 200 ppm) for 24 h. **Fig. S7** The bacterial survival from the bacterial colonies in Fig. 5b. **Fig. S8** Gram, masson trichrome, and hematoxylin–eosin (H&E) and staining of wound tissues after exposure to different treatment (PBS, M-MoS_2_, M-MoS_2_ + Laser, M-MoS_2_ + US, and M-MoS_2_ + Laser + US) for 10 days. **Fig. S9** H&E staining of main organs (heart, liver, spleen, lung, and kidney) in different treatment groups.

## Data Availability

All data of this study are available from the corresponding author on reasonable request.
