# Correction: Metallic phase enabling Mos_2 _nanosheets as an efficient sonosensitizer for photothermal‑enhanced sonodynamic antibacterial therapy

**DOI:** 10.1186/s12951-025-03884-z

**Published:** 2026-01-20

**Authors:** Huizhi Chen, Xiaojun He, Zhan Zhou, Zhikang Wu, Hai Li, Xinsheng Peng, Yubin Zhou, Chaoliang Tan, Jianliang Shen

**Affiliations:** 1 Development of Natural Drugs School of Pharmacy Guangdong Provincial Key Laboratory of Research, Guangdong Medical University, Dongguan, 523808 China; 2 School of Ophthalmology and Optometry School of Biomedical Engineering, Wenzhou Medical University, Wenzhou, 325035 Zhejiang China; 3 College of Chemistry and Chemical Engineering Henan Key Laboratory of Function-Oriented Porous Materials, Luoyang Normal University, Luoyang, 471934 China; 4 Institute of Advanced Materials (IAM) and Key Laboratory of Flexible Electronics (KLoFE), Nanjing Tech University (NanjingTech), 30 South Puzhu Road, Nanjing, 211816 China; 5 Institute of Advanced Materials (IAM) and Key Laboratory of Flexible Electronics (KLoFE), Nanjing Tech University (NanjingTech), 30 South Puzhu Road, Nanjing, 211816 China; 6 Shenzhen Research Institute, City University of Hong Kong, Shenzhen, 518057 China; 7 Wenzhou Institute, University of Chinese Academy of Sciences, Wenzhou, 325001 Zhejiang China

**Correction: Journal of Nanobiotechnology (2022) 20:136**

10.1186/s12951-022-01344-6

 Following publication of the original article (10.1186/s12951-022-01344-6), the authors identified an error in Fig. 4c and Fig. S8. The details are shown as follows.

The images of *S. aureus Only and S. aureus MoS*_*2*_ *Only *of the control were replaced in Figure 4c. Accordingly, the following sentence was updated to follow: “After US (1.0 MHz, 1.5 W cm^−2^, 50% duty cycle) and laser (1064 nm, 1 W cm^−2^) irradiation for 3 min, the bacteriostatic rate of PVP-modified M-MoS_2_ (50 ppm) reached 18% and 20% for *P. aeruginosa* (**43.9%** and **21.4%** for* S. aureus*), respectively (Fig. 4b and d).” The image of Masson Staining of the control was replaced in Fig. S8. The correction of this information does not affect the results and conclusions of this paper. The final Fig. 4 and Fig. S8 are shown as follows:

Incorrect Fig. 4


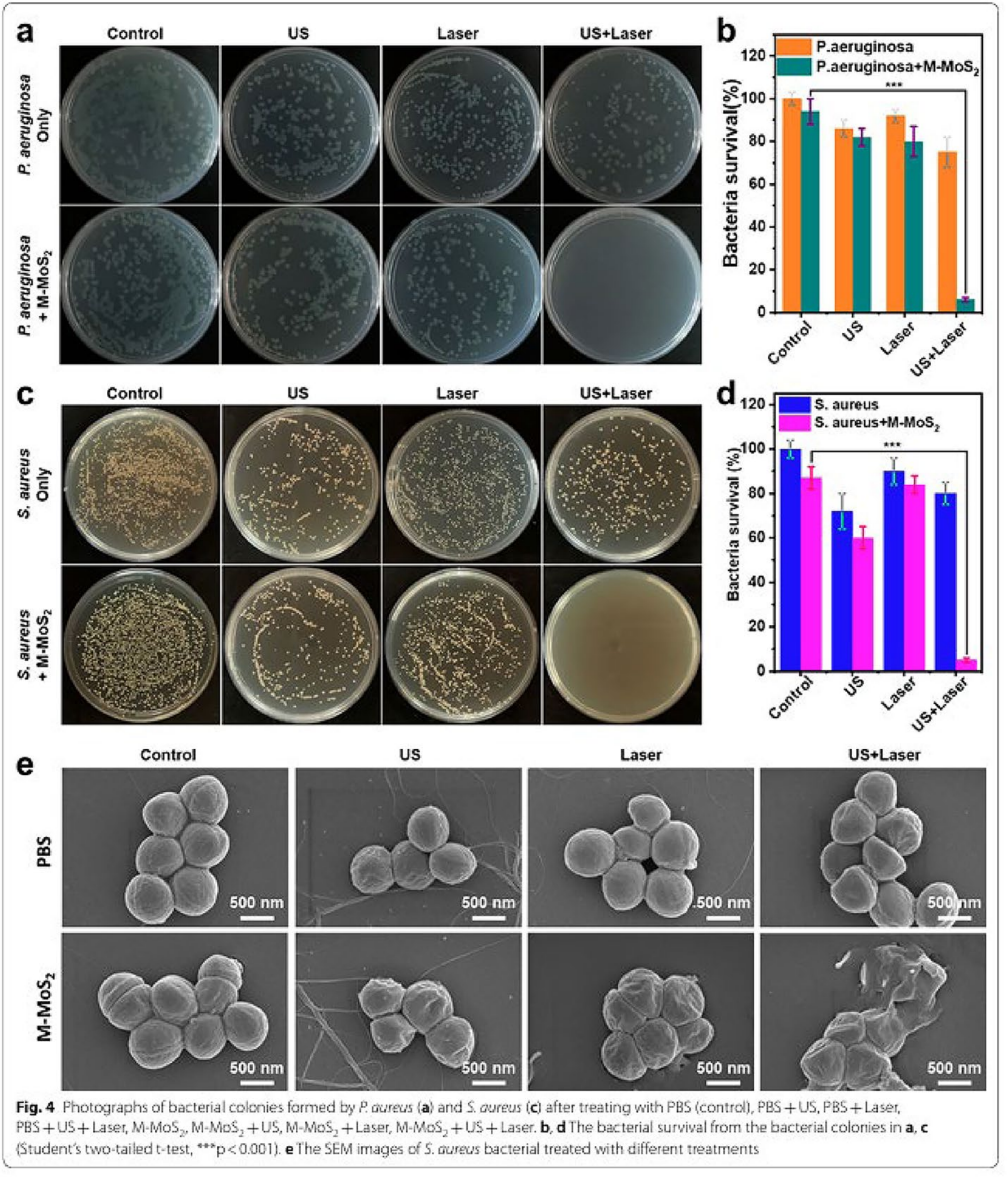


 Correct Fig. 4

**Fig. 4:**
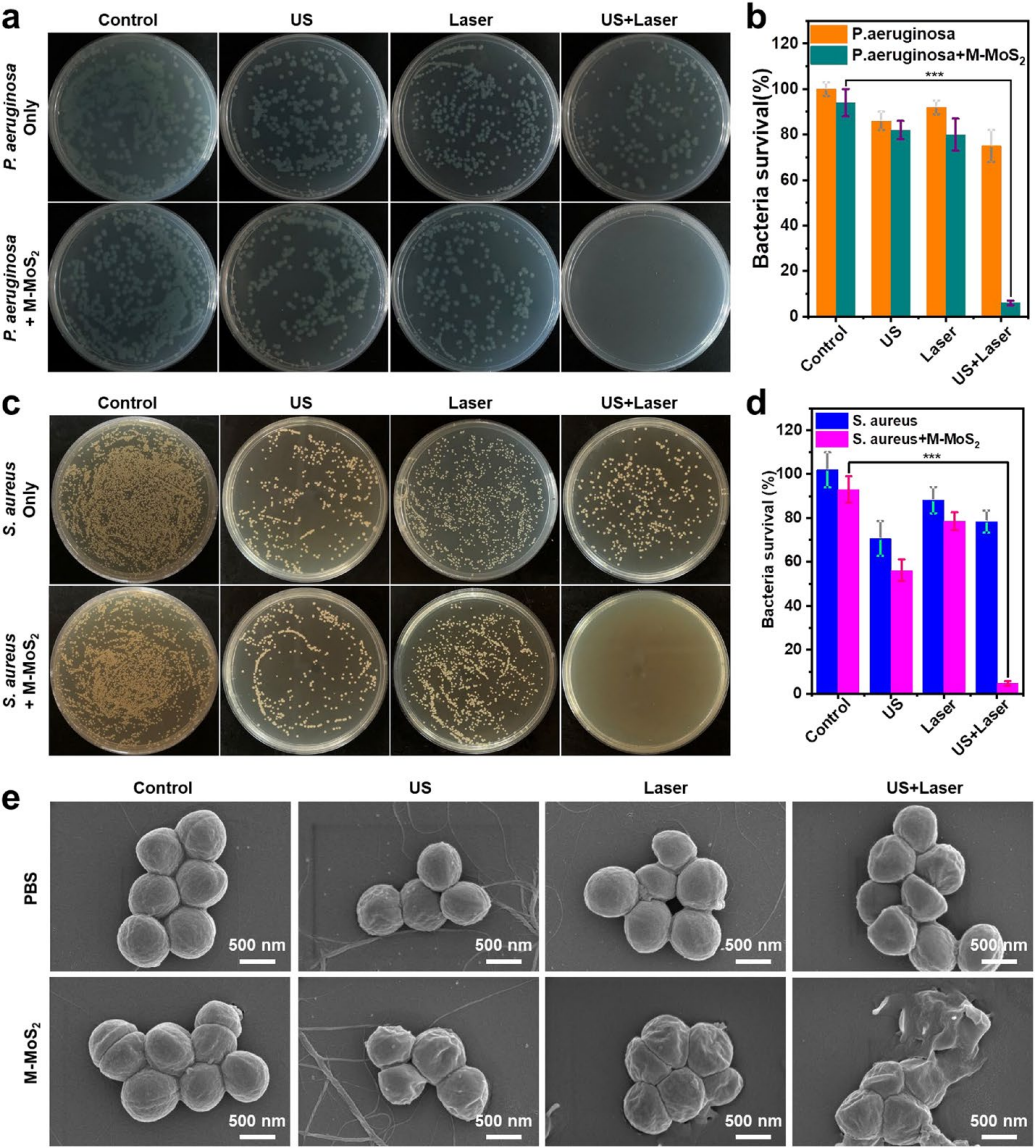
Photographs of bacterial colonies formed by P. aureus (**a**) and S. aureus (**c**) after treating with PBS (control), PBS + US, PBS + Laser, PBS + US + Laser, M-MoS2, M-MoS2 + US, M-MoS2 + Laser, M-MoS2 + US + Laser. **b, d** The bacterial survival from the bacterial colonies in **a, c** (Student’s two-tailed t-test, ***p < 0.001). **e** The SEM images of S. aureus bacterial treated with different treatments

Incorrect Fig. S8


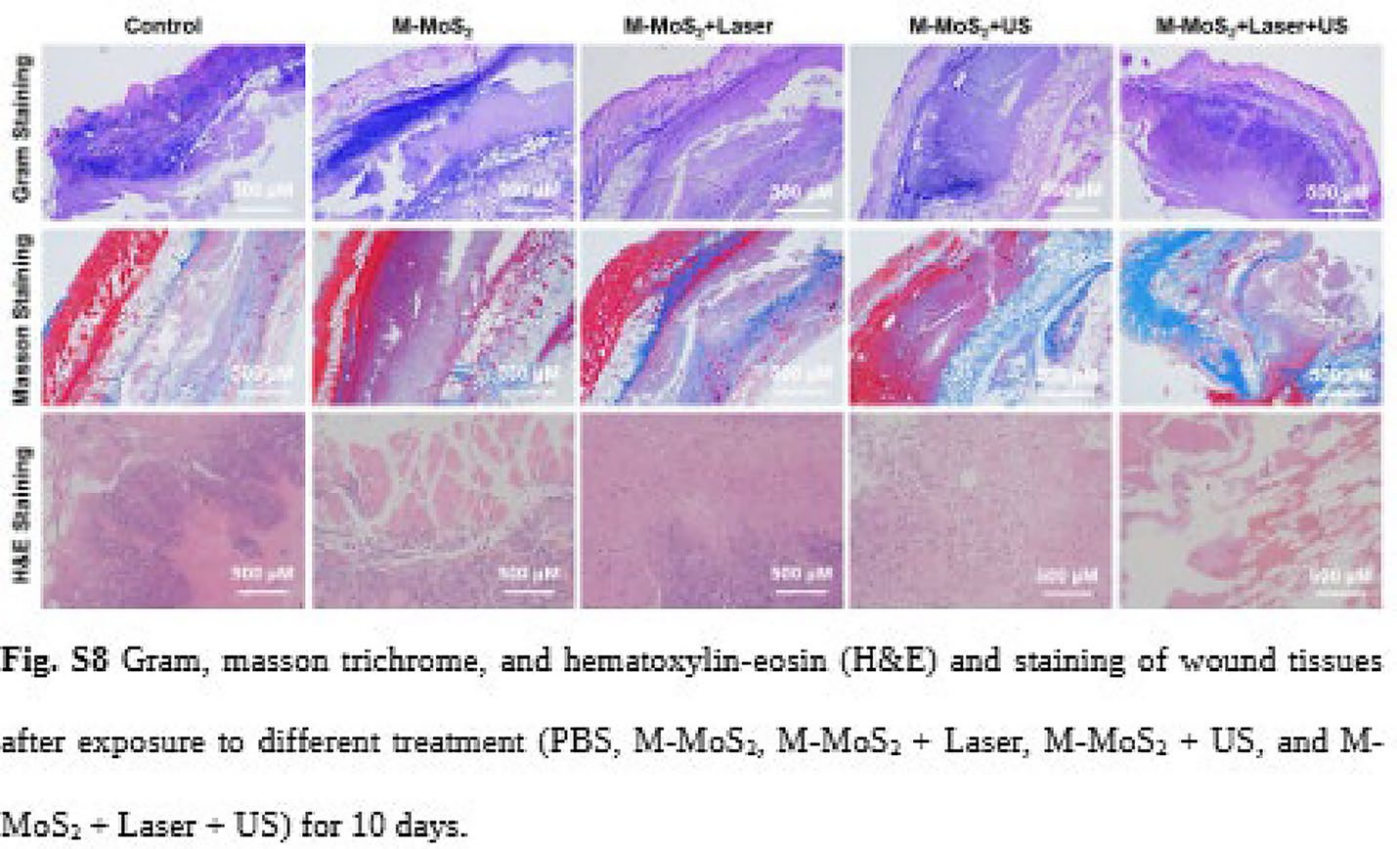


Correct Fig. S8

**Fig. S8:**
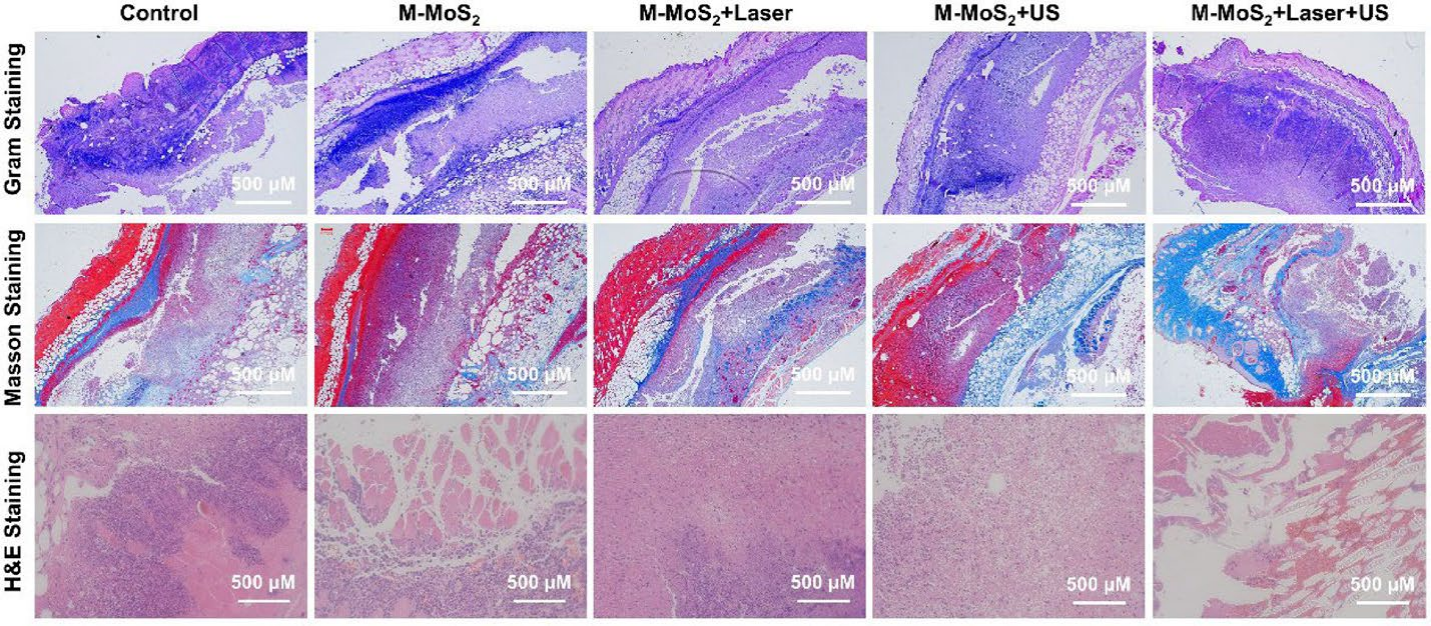
Gram, masson trichrome, and hematoxylin–eosin (H&E) and staining of wound tissues after exposure to different treatment (PBS, M-MoS2, M-MoS2 + Laser, M-MoS2 + US, and M-MoS2 + Laser + US) for 10 days

