# Supplementary material for: Metallic phase enabling MoS2 nanosheets as an efficient sonosensitizer for photothermal-enhanced sonodynamic antibacterial therapy
Source: J Nanobiotechnology. 2022 Mar 15;20:136. doi: 10.1186/s12951-022-01344-6 (PMC8922895; doi:10.1186/s12951-022-01344-6)
Supplement: Supplementary file 1 — Additional file 1: Fig. S1 (a) SEM images of 2H-phase MoS2 bulk crystals and (b) its XRD pattern with simulated reference. Fig. S2 (a) TEM image of S-MoS2 nanosheets and (b) the HRTEM image of a typical S-MoS2 nanosheet. (c) AFM height image of S-MoS2 nanosheets. (d) Atomic-resolution HAADF-STEM image of a typical S-MoS2 nanosheet. (e) The filtered of the marked squire regime in (d) and (f) the corresponding crystal structure of semiconducting 2H-phase MoS2. Fig. S3 (a) UV–vis-NIR absorption spectra of M-MoS2 nanosheets dispersed in water at different concentrations and (b) its corresponding normalized absorbance intensity divided by the characteristic length of the cell (A/L) at different concentrations for λ = 1064 nm. Fig. S4 (a) Thermal images of PVP-modified M-MoS2 and PVP-modified S-MoS2 nanosheets with different concentrations (0, 10, 20, and 50 ppm) under the irradiation by a laser at 1.0 W cm−2 for 6 min. Photothermal heating curves water solutions containing (b) PVP-modified M-MoS2 and (c) PVP-modified S-MoS2 nanosheets at different concentrations (0, 5, 10, 20 and 50 ppm). (d) Photothermal heating curves water solutions containing 50 ppm PVP-modified M-MoS2 irradiated with a laser at different power density. (e) Heating of solution of 50 ppm PVP-modified M-MoS2 and S-MoS2 nanosheets for five On/Off cycles. Fig. S5 (a) The molecular structure of the reaction between RB and active oxygen species. (b,c) UV–vis spectra of RB in absence of and in presence of 50 ppm (b) M-MoS2 and (c) S-MoS2 nanosheets after US treatments with different times. Fig. S6 Relative viabilities of cells (L929) after incubation in PVP-modified M-MoS2 and S-MoS2 nanosheets with different concentrations (0, 5, 10, 20, 40, 80, 100, 120, 150 and 200 ppm) for 24 h. Fig. S7 The bacterial survival from the bacterial colonies in Fig. 5b. Fig. S8 Gram, masson trichrome, and hematoxylin–eosin (H&E) and staining of wound tissues after exposure to different treatment (PBS, M-MoS2, M-MoS2 + Laser, [file 12951_2022_1344_MOESM1_ESM.docx]

***Supplementary Information***

**Metallic phase enabling MoS_2_ nanosheets as an efficient Sonosensitizer for photothermal-enhanced sonodynamic antibacterial therapy**

Huizhi Chen^1,†^, Xiaojun He^2,†^, Zhan Zhou^3,†^, Zhikang Wu^4^, Hai Li^4^, Xinsheng Peng^1^, Yubin Zhou^1,^*, Chaoliang Tan^5,6,^*, Jianliang Shen^2,7,^*

^1^ Guangdong Provincial Key Laboratory of Research and Development of Natural Drugs, and School of Pharmacy, Guangdong Medical University, Dongguan, 523808 P. R. China

^2^ School of Ophthalmology & Optometry, School of Biomedical Engineering, Wenzhou Medical University, Wenzhou, Zhejiang 325035, China

^3^ College of Chemistry and Chemical Engineering, Henan Key Laboratory of Function-Oriented Porous Materials, Luoyang Normal University, Luoyang, 471934, China

^4^ Institute of Advanced Materials (IAM) and Key Laboratory of Flexible Electronics (KLoFE), Nanjing Tech University (NanjingTech), 30 South Puzhu Road, Nanjing 211816, China

^5^ Department of Electrical Engineering, City University of Hong Kong, 83 Tat Chee Avenue, Kowloon, Hong Kong, China

^6^ Shenzhen Research Institute, City University of Hong Kong, Shenzhen, 518057, China

^7^ Wenzhou Institute, University of Chinese Academy of Sciences, Wenzhou, Zhejiang 325001, China

^†^ These authors contribute equally to this work.

* Correspondence: Yubin Zhou (zybresearch@126.com) or Chaoliang Tan ([chaoltan@cityu.edu.hk](mailto:chaoltan@cityu.edu.hk)) or Jianliang Shen (shenjl@wiucas.ac.cn)

**Fig. S1** (a) SEM images of 2H-phase MoS_2_ bulk crystals and (b) its XRD pattern with simulated reference.


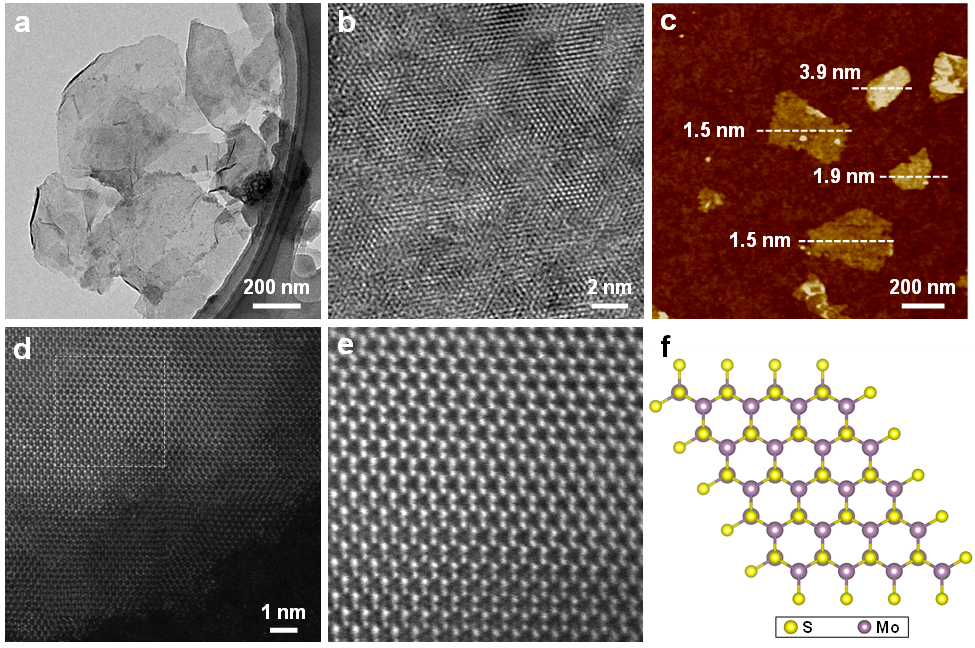


**Fig. S2** (a) TEM image of S-MoS_2_ nanosheets and (b) the HRTEM image of a typical S-MoS_2_ nanosheet. (c) AFM height image of S-MoS_2_ nanosheets. (d) Atomic-resolution HAADF-STEM image of a typical S-MoS_2_ nanosheet. (e) The filtered of the marked squire regime in (d) and (f) the corresponding crystal structure of semiconducting 2H-phase MoS_2_.

**Fig. S3** (a) UV-vis-NIR absorption spectra of M-MoS_2_ nanosheets dispersed in water at different concentrations and (b) its corresponding normalized absorbance intensity divided by the characteristic length of the cell (*A/L*) at different concentrations for λ = 1064 nm.


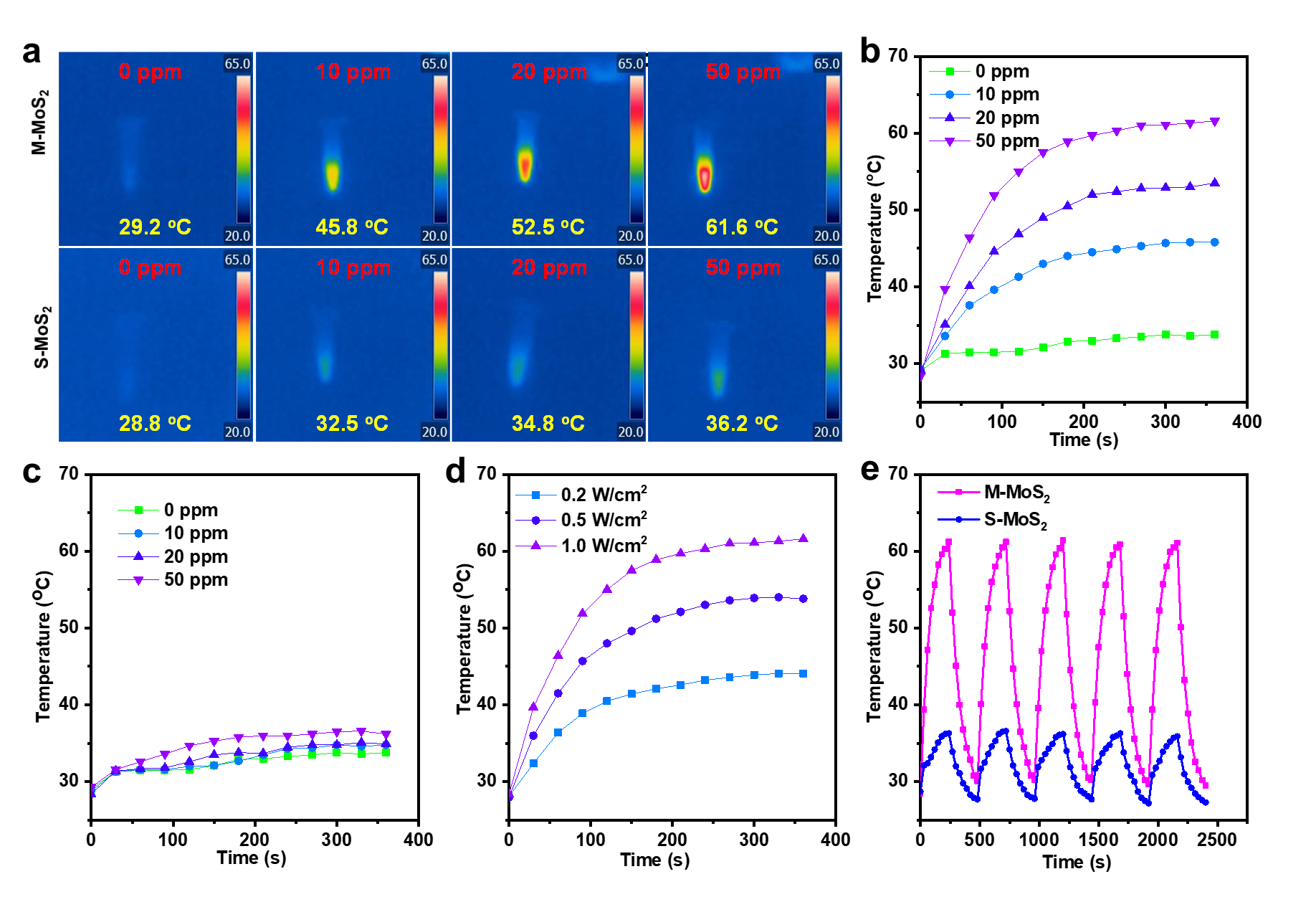


**Fig. S4** (a) Thermal images of PVP-modified M-MoS_2_ and PVP-modified S-MoS_2_ nanosheets with different concentrations (0, 10, 20, and 50 ppm) under the irradiation by a laser at 1.0 W cm^−2^ for 6 min. Photothermal heating curves water solutions containing (b) PVP-modified M-MoS_2_ and (c) PVP-modified S-MoS_2_ nanosheets at different concentrations (0, 5, 10, 20 and 50 ppm). (d) Photothermal heating curves water solutions containing 50 ppm PVP-modified M-MoS_2_ irradiated with a laser at different power density. (e) Heating of solution of 50 ppm PVP-modified M-MoS_2_ and S-MoS_2_ nanosheets for five On/Off cycles.

**Fig. S5** (a) The molecular structure of the reaction between RB and active oxygen species. (b,c) UV–vis spectra of RB in absence of and in presence of 50 ppm (b) M-MoS_2_ and (c) S-MoS_2_ nanosheets after US treatments with different times.

**Fig. S6** Relative viabilities of cells (L929) after incubation in PVP-modified M-MoS_2_ and S-MoS_2_ nanosheets with different concentrations (0, 5, 10, 20, 40, 80, 100, 120, 150 and 200 ppm) for 24 h.

**Fig. S7** The bacterial survival from the bacterial colonies in Figure 5b (Student's two-tailed t-test, **p < 0.01).


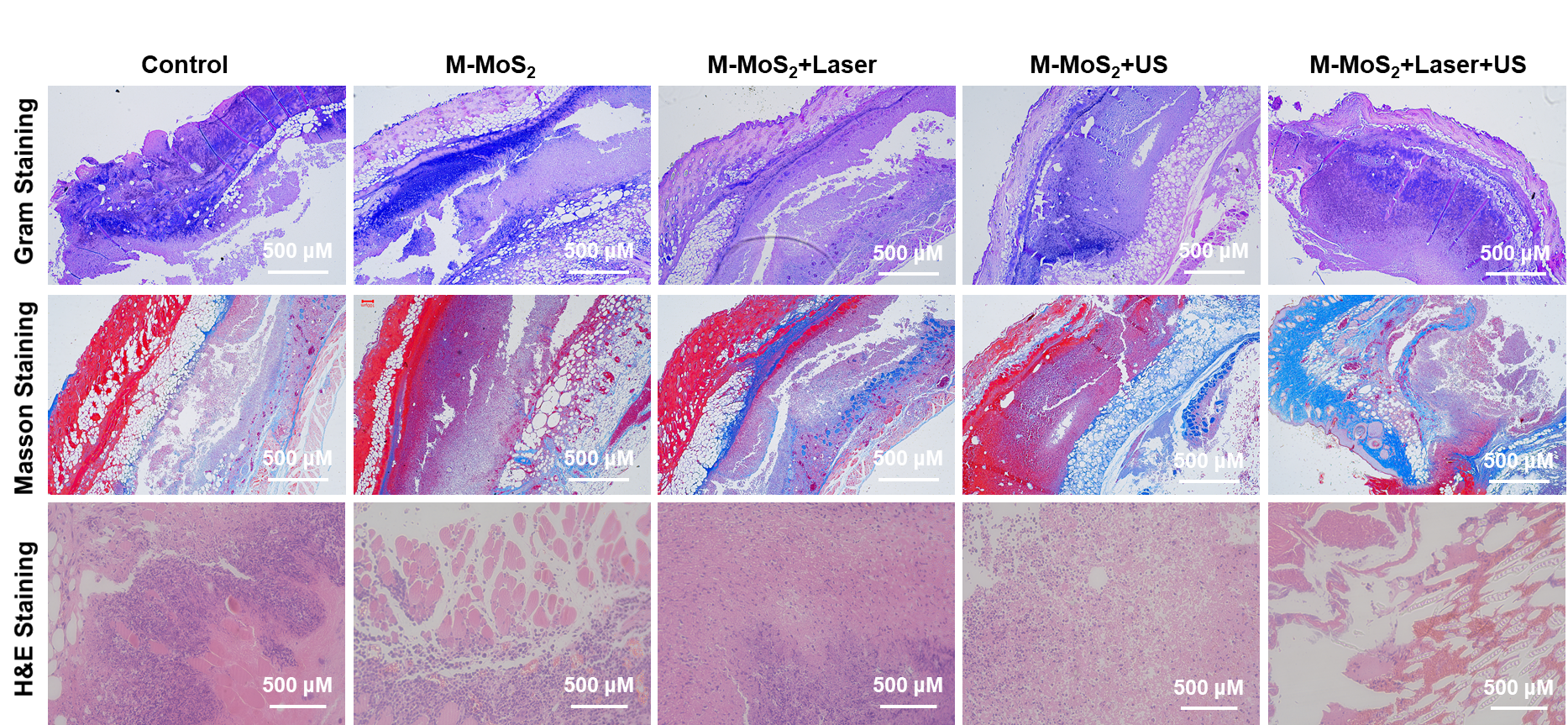


**Fig. S8** Gram, masson trichrome, and hematoxylin-eosin (H&E) and staining of wound tissues after exposure to different treatment (PBS, M-MoS_2_, M-MoS_2_ + Laser, M-MoS_2_ + US, and M-MoS_2_ + Laser + US) for 10 days.


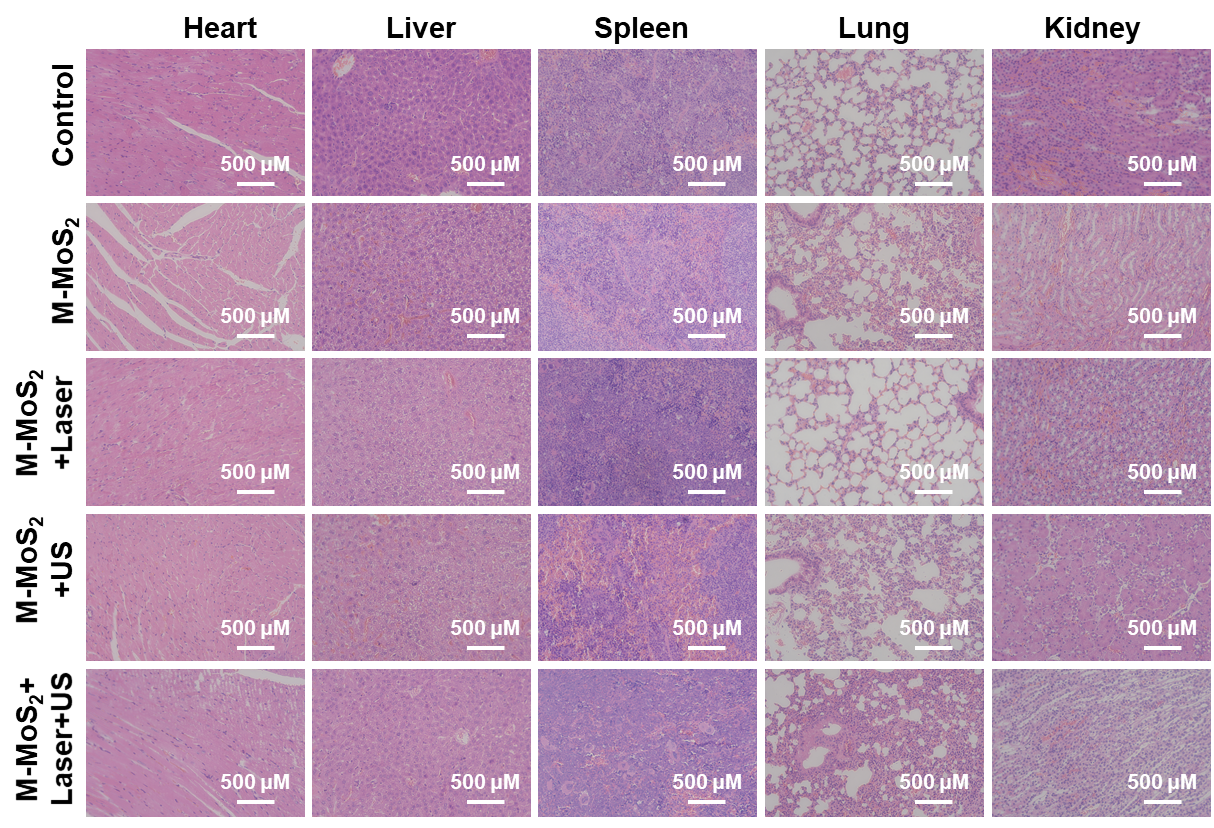


**Fig. S9** H&E staining of main organs (heart, liver, spleen, lung, and kidney) in different treatment groups.
